# National Association of Dental Faculties

**Published:** 1891-09

**Authors:** 


### NATIONAL ASSOCIATION OF DENTAL FACULTIES.

THE Eighth Annual Meeting of the National Association of Dental Faculties was held at Saratoga Springs, commencing Saturday, August 1, 1891; the President, Dr. L. D. Carpenter, Atlanta, in the chair.

The following colleges were represented at the meeting:

*Baltimore College of Dental Surgery.*—R. B. Winder.

*Boston Dental College.*—John A. Follett.

*Chicago College of Dental Surgery.*—Truman W. Brophy.

*Harvard University, Dental Department.*—Thomas Fillebrown.

*Kansas City Dental College.*—J. D. Patterson.

*Missouri Dental College.*—W. H. Eames.

*New York College of Dentistry.*—Frank Abbott.

*Ohio College of Dental Surgery.*—H. A. Smith.

*Pennsylvania College of Dental Surgery.*—C. N. Peirce.

*Philadelphia Dental College.*—Henry I. Dorr.

*State University of Iowa, Dental Department.*—A. O. Hunt.

*University of Michigan, Dental Department.*—J. Taft.

*University of Pennsylvania, Dental Department.*—James Truman.

*Vanderbilt University, Dental Department.*—W. H. Morgan.

*Louisville College of Dentistry.*—James Lewis Howe.

*Indiana Dental College.*—Junius E. Cravens.

*University Dental College.*—George H. Cushing.

*Dental Department of National University.*—James B. Hodgkin.

*Dental Department of Southern Medical College.*—L. D. Carpenter.

*School of Dentistry of Meharry Medical Department of Central Tennessee College.*—G. W. Hubbard.

*University of Maryland, Dental Department.*—Isaac H. Davis.

*Royal College of Dental Surgeons of Ontario.*—J. B. Willmott.

*American College of Dental Surgery.*—T. Clendenen.

Dr. H. B. Noble, of Columbian University, Dental Department, was also present, but not as a delegate.

The University of Denver, Dental Department, was reported upon favorably by the Executive Committee, and was elected a member of the Association, and its representative, W. F. McDowell, took part in the meeting.

The applications of Western Dental College, Kansas City, and Dental Department Medical College of Tennessee, Knoxville, were reported favorably, and under the rules laid over for one year.

On motion, it was directed that the National Association of

Dental Examiners be requested to appoint a committee of five to confer with a similar committee from the National Association of Dental Faculties in regard to matters of mutual interest between the two Associations, their conclusions to be reported back to this meeting. The president appointed as the committee on behalf of the Faculties Association, Drs. Peirce, Winder, Eames, Truman, and Morgan.

The committee subsequently reported the following resolutions as having been agreed to by the conference committees, and recommended that they be confirmed, which was accordingly done.:

*Resolved*, That it is recommended to the State Boards that when a graduate, after examination, has been refused a license and his college requests information as to the causes of his failure to pass the examination, the Boards shall furnish the Faculty with a detailed statement of the subjects and questions on which the applicant has failed.

*Resolved*, That we discountenance the publication by the State Boards of the names of colleges whose graduates have failed to pass.

The committee also reported favorably a communication from the National Association of Dental Examiners, which had been referred to them, as follows:

"As the next session of the colleges marks the commencement of the new plan of three years' college instruction, we recommend that this Association request the National Association of Dental Faculties to require each school to issue each year an announcement containing a list of the students classified in the three grades of seniors, juniors, and freshmen; that this list also in each instance designate the absentees, and that each school be required in the same announcement to publish a list of the graduates of the preceding sessions."

On motion of Dr. Cushing, the term absentee in the foregoing was construed to mean one who for any reason has not attended a full course.

The president being asked to rule upon the resolution adopted last year requiring dissections, decided that it was mandatory.

The following, offered by Dr. Abbott, was adopted by a unanimous vote:

*Resolved*, That any college whose regularly appointed representative fails to sign the constitution within one year from the time of its election to membership shall be dropped from the roll of membership of this Association.

The American College of Dental Surgery, Chicago, was suspended from membership for two years for violation of the rules of the Association in accepting students after the prescribed time and giving them credit for a full term.

A resolution dismissing charges against the Philadelphia Dental College was adopted.

The following were offered and laid over for one year under the rules.

By Dr. Hunt:

*Resolved,* That in case of charges against any college no final action shall be taken until all parties concerned shall have had at least thirty days' notice.

By Dr. Truman:

*Resolved,* That at all future meetings of the National Association of Dental Faculties the delegates shall consist of members of Faculties, and demonstrators will not be received.

By Dr. Fillebrown:

*Voted,* That after June, 1893, the yearly course of study shall be not less than seven months, two months of which may be attendance upon clinical instruction in the infirmary of the school, now known as intermediate or infirmary courses.

By Dr. Hunt:

*Resolved,* That after the session of 1892–93, four years in the study of dentistry be required before graduation.

Dr. Dorr offered a resolution that students who have successfully passed their examinations for advanced standing shall have their certificates given or mailed to them within thirty days after such examinations shall have been completed. Adopted.

The following officers were elected for the ensuing year; W. H. Eames, St. Louis, President; J. D. Patterson, Kansas City, Vice-President; J. D. Patterson, Kansas City, Secretary; H. A. Smith, Cincinnati, Treasurer.

*Executive Committee.*—J. Taft, A. O. Hunt, Frank Abbott.

The president appointed the following standing committees:

*Ad Interim Committee.*—James Truman, Frank Abbott, Thomas Fillebrown.

*Committee on Schools.*—D. R. Stubblefield, J. A. Follett, J. Lewis Howe, J. E. Cravens, S. H. Guilford.

Adjourned to meet at the place of the next meeting of the American Dental Association, on the Monday previous, at 10 o'clock.

